# Size matters: Predator outbreaks threaten foundation species in small Marine Protected Areas

**DOI:** 10.1371/journal.pone.0171569

**Published:** 2017-02-06

**Authors:** Cody S. Clements, Mark E. Hay

**Affiliations:** School of Biological Sciences, Georgia Institute of Technology, Atlanta, United States of America; Department of Agriculture and Water Resources, AUSTRALIA

## Abstract

The unanticipated impacts of consumers in fragmented habitats are frequently a challenge for ecosystem management. On Indo-Pacific coral reefs, crown-of-thorns sea stars (*Acanthaster* spp.) are coral predators whose outbreaks cause precipitous coral decline. Across large spatial scales, *Acanthaster* densities are lower in large no-take Marine Protected Areas (MPAs) and reefs subject to limited human exploitation. However, using a combination of observational and manipulative experiments, we found that *Acanthaster* densities within a network of small, no-take MPAs on reef flats in Fiji were ~2–3.4 times greater inside MPAs than in adjacent fished areas and ~2–2.5 times greater than the upper threshold density indicative of an outbreak. This appeared to result from selective *Acanthaster* migration to the coral-rich MPAs from fished areas that are coral-poor and dominated by macroalgae. Small MPAs can dramatically increase the cover of foundation species like corals, but may selectively attract coral predators like *Acanthaster* due to greater food densities within MPAs or because the MPAs are too small to support *Acanthaster* enemies. As coral cover increases, their chemical and visual cues may concentrate *Acanthaster* to outbreak densities that cause coral demise, compromising the value of small MPAs. An understanding of predator dynamics as a function of habitat type, size, and fragmentation needs to be incorporated into MPA design and management.

## Introduction

The increasing frequency and severity of anthropogenic impacts throughout the global ocean has led to habitat degradation, fragmentation, and trophic downgrading of marine ecosystems worldwide [1, 2]. To counter these trends and promote ecosystem recovery and resilience, Marine Protected Areas (MPAs) are increasingly being established–often with broadly defined goals oriented towards the protection of foundation species (e.g., coral, kelp, seagrass, mangroves, etc.) upon which a broad variety of other species depend [3]. Efforts to establish MPAs have been particularly urgent on tropical coral reefs, which have experienced dramatic declines in coral cover and coral-associated species [4, 5, 6, 7] and in numerous cases have transitioned from structurally complex systems dominated by corals to structurally simplified systems dominated by macrolagae [8, 9].

While the number of MPAs worldwide has steadily increased, MPA design and management strategies are variable, with many no-take MPAs being small habitat fragments embedded within a broader background of exploited, and often degraded, habitat [10]. Indeed, an explicit aim of many MPAs is to aid the rehabilitation of surrounding degraded areas via spillover of adults and export of larvae [11]. There is considerable debate over how size affects MPA performance, but much of this has focused on how size influences protection from human exploitation (e.g., incorporating species’ home ranges and migration) and replenishment of focal species populations (e.g., larval export, recruitment, and spillover) [12, 13, 14]. In contrast, the effects of reserve size on predator densities or behaviors have rarely been addressed, despite the ability of consumers to destabilize species and community-level dynamics–especially if they attack foundation species or ecosystem engineers [1, 15]. Because predators have dramatic direct and indirect impact on community structure and function [1, 16], predicting and mitigating predator-induced disturbances are necessary to safeguard ecosystem integrity and will be increasingly important as global-scale stressors continue to challenge the effectiveness of local management efforts [17, 18].

A major driver of the recent 50% loss in coral cover along the Great Barrier Reef and on reefs throughout the tropical Pacific is predation by the crown-of-thorns sea star (*Acanthaster* spp.) [5, 7], which exhibits population outbreaks that can reduce live coral over vast areas and can lead to the ecological collapse of entire reef systems [19]. *Acanthaster* outbreaks are hypothesized to occur via several mechanisms, including (*i*) reduced population constraints (e.g., predation) that contribute to one or successive mass recruitment events and/or (*ii*) concentrated aggregations of foraging adults (for review, see [20]). *Acanthaster* adults use a combination of chemical and visual sensory cues to navigate toward preferred corals [21, 22, 23], and during outbreaks, have been shown to aggregate on corals being eaten by conspecifics [22] and move en masse from areas of depleted coral to unexploited reef tracts in search of food [19, 24]. There is also correlative evidence across large spatial scales that limited or restricted fishing is associated with low densities of *Acanthaster*–hypothetically due to the maintenance of intact food webs that exert top-down control on *Acanthaster* populations [25, 26]. However, despite these correlations over large areas [25] and long time periods [26], the identity of critical predators and the life-stage of *Acanthaster* on which they feed remain unknown, and therefore speculative as a mechanism of population control.

Retention of food-web connections, along with other fisheries and conservation benefits, have been touted in the literature and used to advocate for MPAs [3, 27], which are now one of the most widespread management tools used by coastal communities throughout the Pacific [28]. Despite their general success [29, 30], some MPAs appear ineffective and can even hasten degradation of remaining critical habitat if they lead to unexpected consumer impacts on foundation species [15]. Studies from terrestrial systems emphasize that habitat fragmentation can lead to mesopredator outbreaks via reduced top-down and bottom-up population constraints [31, 32], but these insights have received limited attention in planning and management of MPAs, especially as a function of size and of being embedded within increasingly fragmented and degraded marine ecosystems. Most MPAs are small (< 1.0 km^2^) [10]–with management focused almost solely on various forms of fishing restrictions (e.g., permanent, partial or periodic restrictions) [28]. Here, we provide evidence that small reserves can be at special risk for predator (*Acanthaster* spp.) outbreaks and suggest that the probability of outbreak densities may increase as conservation succeeds at increasing coral cover and thus food for, and attraction of, *Acanthaster*.

## Materials and methods

### Ethics statement

Research was approved by the Fijian government and the Korolevu-i-Wai Environment Committee, which oversees management of nearshore marine resources where we conducted our research. Fieldwork was performed in accordance with the ethical regulations of the Georgia Institute of Technology and Fijian Law.

### Study area

**Fig 1:**
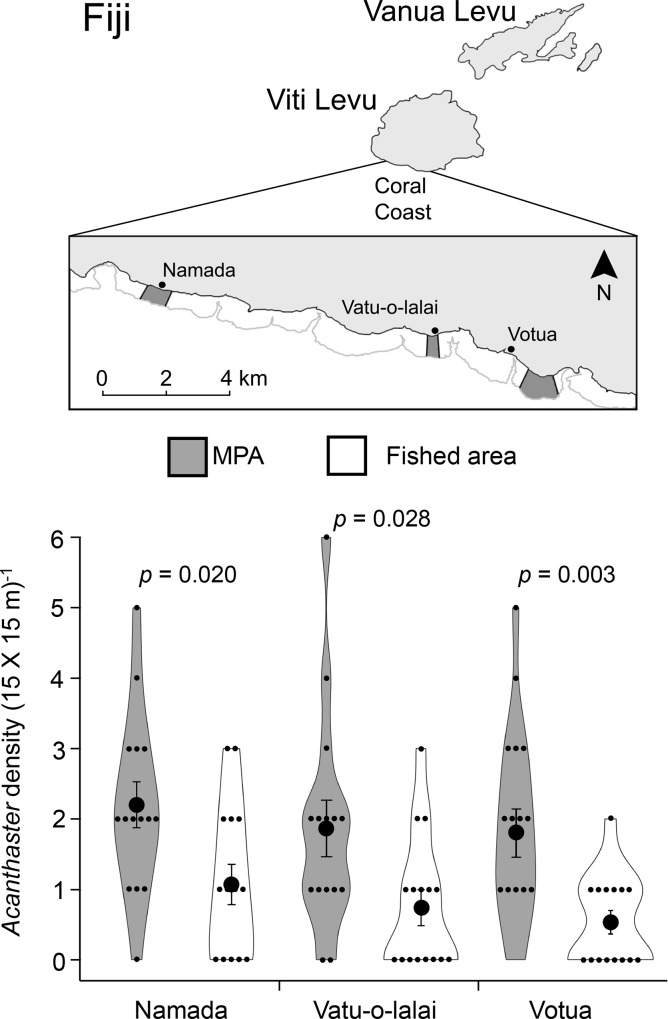
Mean *Acanthaster* density is ~2–3.4 times greater within Marine Protected Areas (MPAs) than adjacent fished areas. (Top panel) Village and MPA locations along the coast of Viti Levu, Fiji. Dark gray sections represent the MPAs at each site. (Bottom panel) Violin plots depicting the mean ± SE *Acanthaster* density (large black dots and error bars), the frequency of plots with differing densities of *Acanthaster* (the enclosed areas), and each individual plot as a function of Acanthaster counted in that 15 X 15 m plot (small black dots) within MPAs (dark gray) and adjacent fished areas (white) at each village (n = 15 quadrats reef^-1^ location^-1^). Data for each pairwise comparison were analyzed using a generalized linear model (GLM) with a Poisson distribution (Votua) or quasi-GLM models (Namada and Vatu-o-lalai).

This study was conducted within paired fished and no-take MPAs on reef flats (depth of ~0–2 m at low tide and ~1–3+ m at high tide) adjacent to Namada, Vatu-o-lalai, and Votua villages along the Coral Coast of Viti Levu, Fiji (18° 13.059’S, 177° 42.979’E) (Fig 1). Paired areas were located within an 11 km stretch of fringing reefs that are separated by a series of deep-water channels. MPAs within this reef system are small (0.45–0.78 km^2^) and separated by ~2.6–10 km. MPAs exhibited high coral cover (~38–56%) and low macroalgal cover (~1–3%) on hard substrates [33, 34], as well as higher biomass and diversity of herbivorous and piscivorous fishes often targeted by artisanal fishers [34, 35]. Conversely, adjacent fished areas were relatively degraded with low coral cover (4–16%), high macroalgal cover (~49–91%) [33, 34], and low biomass and diversity of herbivorous and piscivorous fishes [34, 35].

### *Acanthaster* cf. *planci* density

*Acanthaster* density was quantified in paired MPAs and fished areas using 15×15 m quadrats (*n* = 15 reef^-1^ location^-1^) that were non-overlapping and distributed haphazardly within the reef flat of each area. Surveys entailed a single snorkeler carefully searching for *Acanthaster* within and under rock ledges and coral colonies within each quadrat for five minutes, especially near areas with obvious signs of *Acanthaster* feeding. *Acanthaster* abundance data violated parametric assumptions, so differences between paired MPAs and fished areas were evaluated using Wilcoxon rank-sum pair-wise comparisons.

### Experimental tagging study

To evaluate how tagging might affect *Acanthaster* behavior, we conducted preliminary experiments comparing righting ability and feeding behavior of tagged and untagged *Acanthaster* (*n* = 10 individuals treatment^-1^) that were caged on the reef flat of Votua’s MPA. Ten individuals were each tagged by inserting five plastic tag fasteners at the base of individual arms near the oral disk (S1 Fig), and all individuals were held in individual cages on the reef flat for the 7-day duration of this experiment. Two days were allowed for tag acclimation among the treatment group before experiments were conducted. Righting ability was assessed on days 3 and 7 post-tagging by flipping individuals onto their aboral surface and measuring the time required to right themselves onto their oral surface. This was repeated three times for each individual with a 1-minute rest interval between trials. Prior to analysis, data were log transformed and tested for homogeneity of variance using Bartlett’s test. Mean righting times within and between days were compared using a two-way ANOVA. Individuals were also offered two small fragments of the coral *Montipora hispida* (~8–10 cm length) on days three and five post-tagging to assess the effects of tagging on feeding behavior. Comparisons of whether the corals offered were either both eaten or both not eaten within 24 h were conducted using a Fisher’s exact test (there were no cases of only one coral being eaten).

To test whether *Acanthaster* selectively migrated into the MPAs versus the fished areas, 120 adults of 36 ± 2 cm diameter (from the tips of opposite arms) were collected from the MPAs and adjacent fished areas of reefs flats near Votua, Vatu-o-lalai, and Namada villages, with 20 individuals collected from within and 20 from outside the MPAs at each village site (40 individuals village^-1^ site^-1^). Each individual was tagged with five plastic tag fasteners between the base of individual arms, and labeled flagging tape was attached to the end of each tag fastener to aid in location and identification (S1 Fig). Individuals were then enclosed within cages located along the MPA border perpendicular to the coastline at each site (20 individuals border^-1^ location^-1^) for 48 h to allow for tag acclimation. Upon release, individuals’ movements were monitored at 24 h intervals for four to eight days by physically locating each individual and recording its location via GPS (Garmin GPS 76CSX). GPS coordinates of individual *Acanthaster* positions were imported into ArcMAP (Version 10.3.1), and the Geospatial Modeling Environment extension (Version 0.7.4.0) was used to calculate individuals’ initial and final directions of movement relative to their release point along their respective MPA border, as well as each individual’s net displacement between consecutive days (S1 Fig). The angular directions of individuals’ positions relative to the MPA border were plotted as circular data and together tested for circular uniformity against an alternative that presumes a specified angle (e.g., 90°) using Batschelet’s modified Hodges-Ajne test. This analysis was conducted for both the first and final relocation of each individual because initial orientations are more suitable for evaluating patch detection capabilities [36, 37].

To determine whether an individual’s origin influenced their movement direction, we compared relocations, both pooled across all villages and individually for each MPA border, of *Acanthaster* collected from the MPAs and fished areas. We also characterized the path directionality of individual sea star movements at each border where data from two or more consecutive movements (relocations on two+ days in a row) were available using the ratio of *D* (the net displacement from initial to final position in the path) to *W* (total distance traveled between days) [38, 39]. A *D*:*W* ratio of 1 represents an individual exhibiting uniformly directional movement (i.e., straight-line path). Values > 0.7 are considered highly directional, > 0.5 partially directional, and < 0.5 undirected [38, 39].

### MPA border benthic surveys

Surveys of benthic community composition were conducted to assess habitat differences inside and outside of each MPA border and the relationship between coral cover and *Acanthaster* displacement at each border. Surveys used 40 m point intercept transects (*n* = 20 transects border^-1^ MPA^-1^, points at 0.5 m intervals, 1,600 points border ^-1^) that were non-overlapping (mean distance between transects = ~12 m) and oriented parallel to the coastline, with the midpoint (20 m) of each transect positioned on the MPA border (20 m within the MPA and 20 m within the fished area) (S1 Fig). Benthic data from within and outside each MPA border were square root transformed if needed, and analyzed using t-tests. When benthic data could not be transformed to meet parametric assumptions, the original count data were used and analyzed with quasi-GLM models. To test for correlations between coral cover and *Acanthaster* movement among sites, coral cover along the transect at the site of each individual’s release as well as pooled coral cover by MPA border were each, separately, linearly regressed against the displacement between consecutive days exhibited by the individual sea star at that location and the mean displacement along each individual MPA border, respectively.

## Results

We found that *Acanthaster* densities within MPAs (~80–98 ha^-1^) were ~2–3.4 times greater than within fished areas (~23–47 ha^-1^; *p* ≤ 0.030, Fig 1), as well as ~2–2.5 times greater than the upper threshold density indicative of an outbreak (40 individuals per hectare [24]). Our tagging methods affected neither righting times (*p* = 0.190) nor frequencies of feeding (*p =* 1.000) for *Acanthaster*; there also was no effect of assessing these behaviors on days three or seven post tagging (*p* ≥ 0.719, S1 Table).

**Fig 2:**
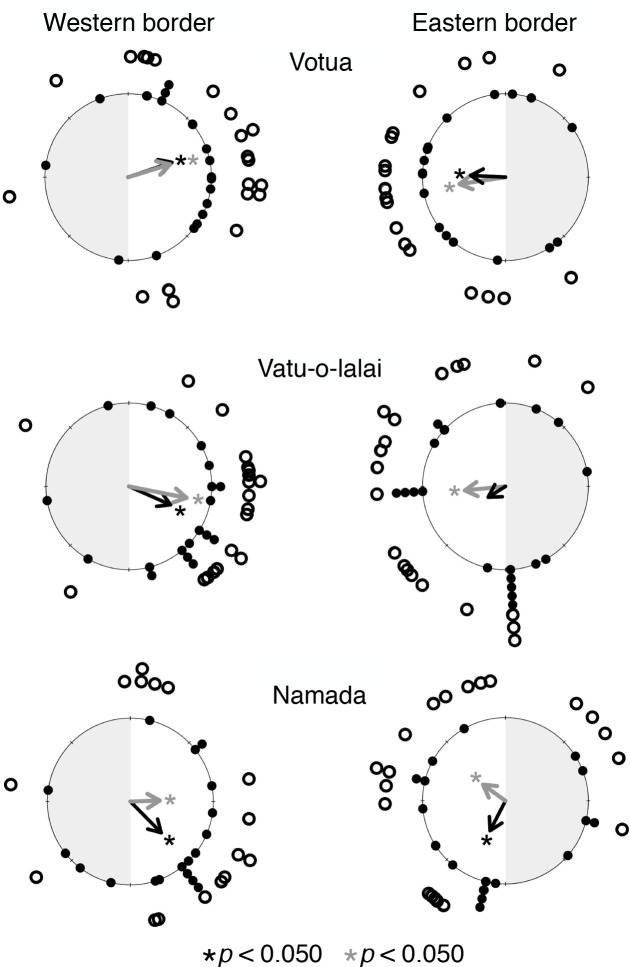
*Acanthaster* move selectively to Marine Protected Areas (MPAs). Movement directions of initial (solid dots) and final (open dots) *Acanthaster* relocations from release points at MPA/non-MPA borders on each side of the MPA at each of the three villages. Arrows represent the resultant vector (R) for initial (black) and final (gray) relocations. * and * indicate a significant difference between *Acanthaster* movement towards MPAs (white region) rather than fished areas (shaded region) (Modified Hodges-Ajne test, *p* < 0.050).

When *Acanthaster* were released along MPA borders, their directions of initial movement were significantly biased toward the MPA for five of the six borders (*p* < 0.050, Fig 2), and suggestive of an MPA preference in the remaining contrast. Approximately 73% of all individuals released and relocated (85 of 116) moved to the MPA, a pattern that was consistent regardless of whether *Acanthaster* were originally collected from the MPAs or fished areas (*p* > 0.656, S2 Table). Similarly, final movement positions were significantly biased toward MPAs for all six contrasts (*p* < 0.050, Fig 2). The ratio of net displacement (*D*) to total displacement between consecutive days (*W*) indicated that *Acanthaster* movement paths exhibited considerable directionality at five of the six MPA borders (*D*:*W* = 0.453–0.717, S3 Table).

**Fig 3:**
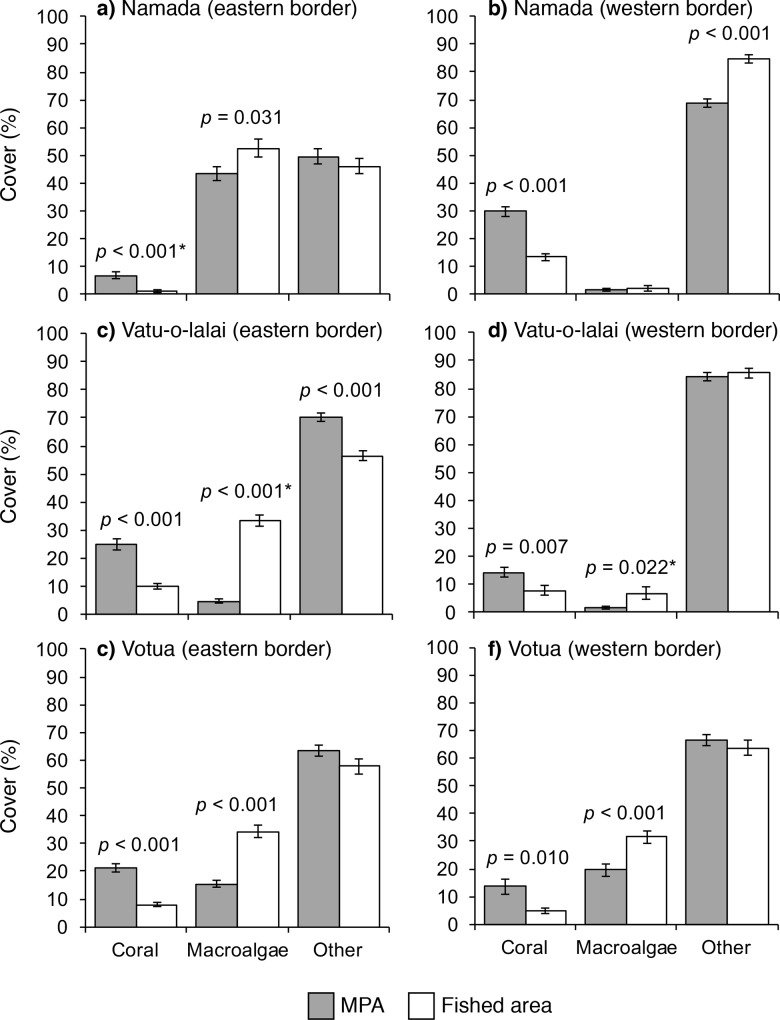
Reef habitat differs immediately inside vs. outside MPAs. Comparisons of benthic cover (mean % ± SE) 20 m inside (black) and 20 m outside (gray) of MPA borders perpendicular to the coastline at Namada, Vatu-o-lalai, and Votua villages (*n* = 20 transects border^-1^ location^-1^). The category “Other” includes dead coral, rock, rubble/sand, and uncommon benthic organisms (e.g., zooanthids, soft coral). Asterisks after *p*-values indicate comparisons analyzed with quasi-GLM models.

**Fig 4:**
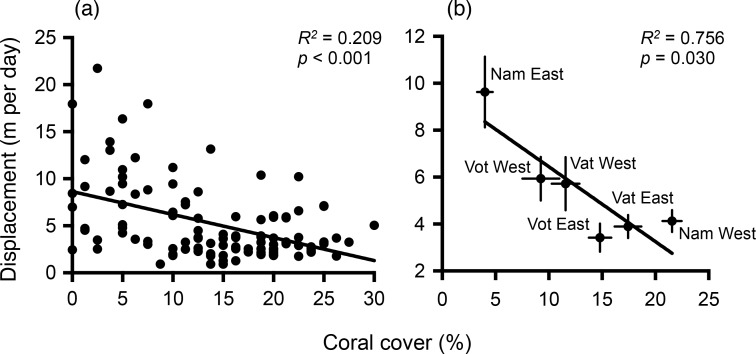
*Acanthaster* displacement is negatively correlated with local coral cover. (a) Relationship between individual *Acanthaster* displacement between consecutive days (m day^-1^) and coral cover (%) at each individual’s release location along MPA borders. (b) Relationship between coral cover (mean % ± SE) and *Acanthaster* displacement between consecutive days (m day^-1^; mean ± SE) when pooled by MPA border. See Fig 1 for village site names. Coefficients of regression (*R*^2^) and *p*-values are indicated in the graph. Two data points with extreme *Acanthaster* displacement values (*y*_1_ = 42.65 m, *y*_2_ = 34.39 m) at low coral cover (*x*_1_ = 0%, *x*_2_ = 11.25%) were excluded from analyses after performing an outlier analysis (Jackknife distances) using JMP (Version 11.0.0).

Benthic community composition commonly differed immediately within versus outside MPAs, with coral and macroalgal cover exhibiting the most frequent significant differences across MPA borders (Fig 3). Coral cover 20 m within MPA borders was 80–440% greater than in the 20 m outside MPA borders, while macroalgal cover was 20–610% greater immediately outside versus inside the MPAs; differences were even more pronounced toward the centers of each area [34]. *Acanthaster* rates of displacement were negatively correlated with mean coral cover along each border, both when plotted by individual *Acanthaster* (*R*^*2*^ = 0.209, *p* < 0.001) and when pooled by MPA border (*R*^*2*^ = 0.756, *p* = 0.030; Fig 4).

## Discussion

Our findings suggest that at small scales, common MPA benefits (e.g., increased coral cover) may attract predators such as *Acanthaster*. *Acanthaster* were 2–3.4 times as abundant within the coral-rich MPAs, exhibiting densities similar to those that have caused extensive coral decline (e.g., >50% [40]), and lead to cascading effects on reef structure and associated species [19]. This unanticipated pattern may provide an important lesson for the management of MPAs across the Pacific, as the overwhelming majority of tropical Pacific MPAs are small (<0.5 km^2^ [28]) and like those in this study, are situated within a background of increasingly degraded reef habitat [5]. Given the widespread use of small MPAs as management tools [10, 28] and the destructive impacts that *Acanthaster* feeding can have on coral reefs at the densities documented here [19, 40], our study highlights the need to consider how the size and placement of MPAs influence their susceptibility to *Acanthaster* outbreaks, and whether the probability of outbreaks increases with MPA success (enhanced coral cover, the foundation species for this system). Despite these high densities of *Acanthaster*, corals are still abundant in the MPAs we investigated [33, 34]. This may be due to *Acanthaster* densities increasing recently and not yet strongly suppressing coral cover or due to coral growth rates on these shallow, turbulent, and well-lit platforms being high enough to generate positive net growth despite high rates of consumption.

Our findings contrast with previous studies where *Acanthaster* densities were reduced in large MPAs or areas subject to limited fishing pressure [25, 26]. Our patterns may differ from these earlier studies due to (*i*) small MPAs having greater perimeter to area ratios that facilitate increased movement of *Acanthaster* into coral-rich MPAs, (*ii*) habitat disparities between coral-rich MPAs and surrounding degraded reefs that enhance *Acanthaster* recruitment and immigration to coral-rich MPAs, (*iii*) differences in critical consumers or processes between MPAs located on shallow (~1–3+ m) reef flats like those we studied vs. reefs from previous studies (~7m in depth or greater), or (*iv*) large MPAs supporting critical consumers or processes that are not sustainable in the small MPAs we studied. That said, it is critical to note that previous studies assumed that predation suppressed *Acanthaster* densities in large MPAs or areas with reduced fishing pressure [25, 26], but this assumption was not directly tested. Neither the identities of the critical predators of *Acanthaster* nor the life stage at which predation could control *Acanthaster* have been determined. Regardless, it is evident from our tagging and density data that *Acanthaster* can selectively migrate into the coral-rich MPAs vs. the coral-poor fished areas and that predation within these reef systems is insufficient to reduce *Acanthaster* numbers to densities below those capable of causing considerable damage to coral communities. These findings highlight an important risk for the many small MPAs embedded within increasingly fragmented and degraded reef ecosystems.

Predator outbreaks may occur in small MPAs due to increased resource availability as the MPAs become effective and enhance the abundance of foundation species that serve as attractive foods for consumers [15]. Our findings build on a small, but growing, body of evidence that consumer attraction may be a critical vulnerability for effective management, as similar scenarios have been documented in other systems, including attraction and overgrazing of seagrass MPAs by sea turtles [15] and plant community regime shifts due to elephant aggregations in African reserves [41, 42]. On coral reefs, this phenomenon may be especially problematic if degraded areas near MPAs serve as nurseries for predators such as *Acanthaster*. For *Acanthaster*, degraded areas surrounding reserves have abundant coral rubble (into which juvenile *Acanthaster* selectively recruit [43]) and considerable abundance of crustose coralline algae, a favored food of juvenile *Acanthaster* (for review, see [20]). Increased juvenile survival in these degraded reef areas followed by selective migration to coral-rich MPAs could contribute to the high *Acanthaster* densities we documented within MPAs.

A second possibility, or additional contributor, to the density difference we noted is that small, fragmented systems may lack top predators, sometimes allowing mesopredators like *Acanthaster* to escape consumer control [31, 32, 44]. Predatory fish biomass was low in both the small MPAs and the fished areas we investigated [35, 45, 46] and is comparable to, or lower than, the biomass of predatory fishes on reefs previously associated with high *Acanthaster* population densities [25]. Lower coral abundance in fished areas may also reduce predation on larval and juvenile *Acanthaster* by coral-associated planktivorous fishes, which have been shown in laboratory trials to prey upon *Acanthaster* larvae [47]. While the identity and roles of predatory fishes controlling *Acanthaster* densities in the wild are largely unknown [20, 25, 26], it is plausible that our predator-depauperate reefs are incapable of exerting top-down control on *Acanthaster* (e.g., predation during vulnerable pre-reproductive stages [26]).

Regardless of what processes normally control *Acanthaster* densities, our tagging data show that migration of adult sea stars from degraded areas could lead to outbreak densities within the coral-rich MPAs. *Acanthaster* consistently moved towards the MPAs at rates proportional to local coral density; a behavior consistent with outbreak scenarios where sea stars migrate from areas of low coral abundance and aggregate on remaining coral patches [19, 48, 49]. However, rather than aggregative behavior induced by recent coral decline, data from the MPAs we studied suggest that increases in live coral following MPA establishment [33, 34, 45] are producing “food hotspots” that attract sea stars from surrounding overfished areas to form ‘spot’ outbreaks [50]. Greater herbivore control of macroalgae within MPAs [34] may further exacerbate this hotspot effect because macroalgae suppress *Acanthaster* feeding on adjacent corals [51], resulting in corals within the MPAs being not only more abundant and more attractive, but also more accessible to *Acanthaster* than corals in the degraded, seaweed-dominated areas surrounding the MPAs. Thus, common benefits of MPAs may become liabilities if reef spatial dynamics, consumer movements, and species interaction networks are not considered in a community context that extends beyond reserve borders.

While many outbreak densities of *Acanthaster* appear to occur following massive recruitment events [20, 52], this did not appear to be the process generating outbreak densities in our sites. We did not note high densities of *Acanthaster* in the fished areas or on deeper portions of adjacent reefs. Rather than resulting from boom and bust cycles, the high densities noted in the MPAs we studied appeared to result from lower chronic densities of *Acanthaster* aggregating in the food hot-spots generated within MPAs. Thus, these localized outbreak densities seem to be generated by different processes [48, 53] and to occur on different temporal and spatial scales than outbreaks noted in many previous investigations (for review, see [20]).

Optimizing local-scale management can provide a critical buffer for ecosystems subject to an increasing array of local and global disturbances [54]. Our study highlights a shortcoming of basic extraction restrictions if these are not integrated with issues of scale, migration, and food web dynamics. Across the Pacific where customary ownership and governance of marine resources occurs at a local scale, small MPAs are among the most common strategies used to manage coral reef ecosystems [28]. When enforced, they can produce remarkably positive effects [27, 29, 30], but as positive outcomes accumulate, this success may concentrate coral predators and endanger MPA resilience. An appreciation for mechanisms generating predator outbreaks needs to be included in the conceptual toolkit of MPA managers. This is particularly relevant to small, locally-managed MPAs where control of *Acanthaster* by physical removal, injections, or other means is likely feasible [55]. However, most MPA management strategies are limited to fishing restrictions that vary in scope and duration (e.g., permanent, partial, or periodic restrictions) [28] and are likely incapable of facilitating adequate biological control of *Acanthaster*. While protection from extraction may be conferring other benefits commonly expected from MPAs, the concern is that without active management of predators like *Acanthaster*, current schemes may promote situations where predation threatens the foundation species upon which MPA success is built. This could compromise gains that have been made since reserve establishment, as well as those expected for the future.

## Supporting information

S1 Fig Experimental tagging and monitoring of *Acanthaster*. (a) Diagram of *Acanthaster* tagging and (b) photograph of a tagged *Acanthaster*. (c) Diagram of experimental design for tagged *Acanthaster* released along each MPA border and benthic surveys conducted along each MPA border. See key below diagram for symbol identification.
(TIF)

S1 Table Experimental tagging did not affect *Acanthaster* righting ability. Two-way ANOVA on the effect of tagging on righting time of *Acanthaster* after 2 and 7 days. All data were log transformed. Bartlett test for homogeneity of variances (*F* = 0.883, *p* = 0.347).
(TIF)

S2 Table Movements of *Acanthaster* originating from the MPAs or fished areas into the MPA or fished areas at each MPA border. Comparisons between *Acanthaster* of different origins tested with Fisher’s exact test.
(TIF)

S3 Table *Acanthaster* movement paths exhibited considerable directionality at five of the six MPA borders. *Acanthaster* net displacement (m day^-1^; mean ± SE), displacement between consecutive days (m day^-1^; mean ± SE), and *D*:*W* ratio (mean ± SD) at MPA border locations.
(TIFF)
