# Supplementary figures and images for: Size matters: Predator outbreaks threaten foundation species in small Marine Protected Areas
Source: PLoS One. 2017 Feb 6;12(2):e0171569. doi: 10.1371/journal.pone.0171569 (PMC5293237; doi:10.1371/journal.pone.0171569)

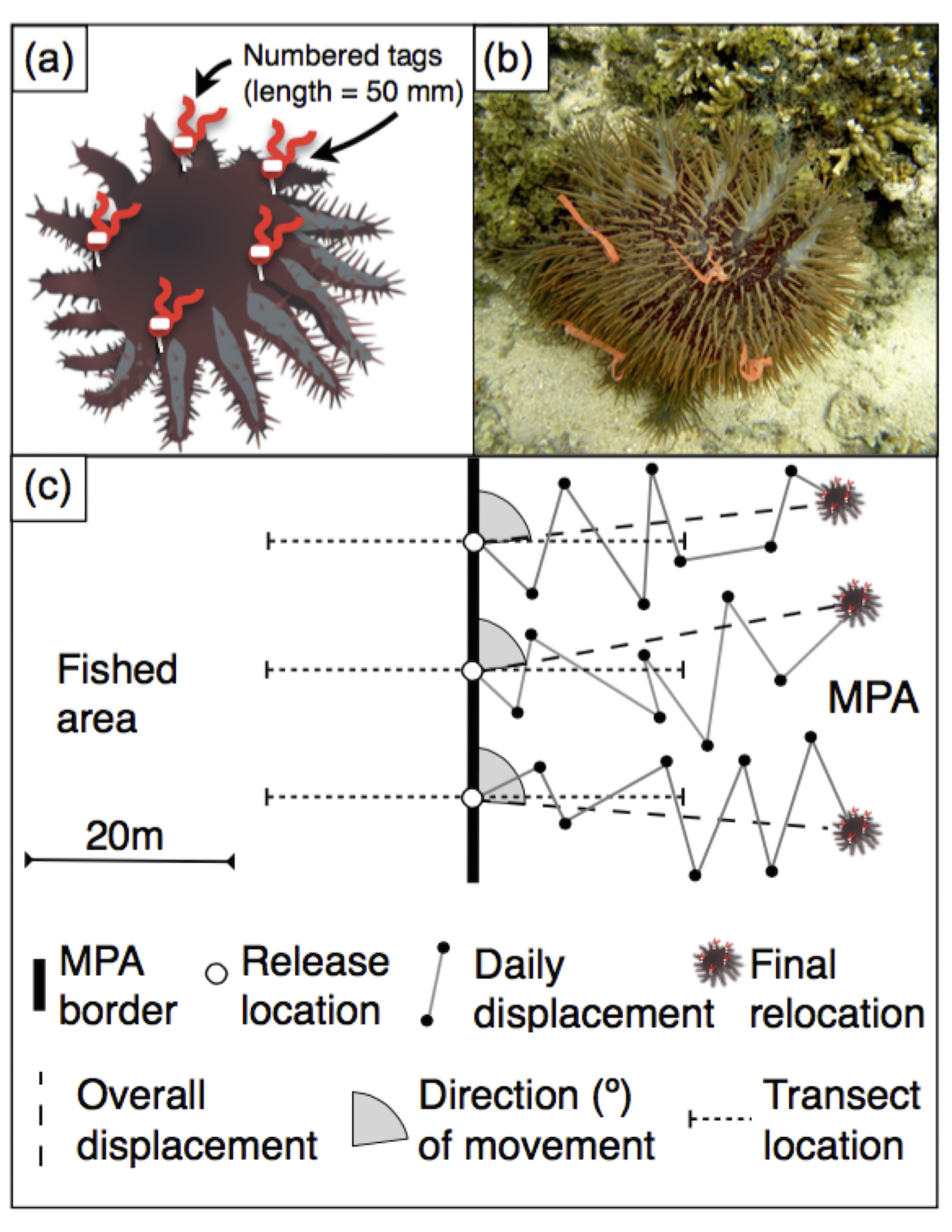

Supplement: S1 Fig — (a) Diagram of Acanthaster tagging and (b) photograph of a tagged Acanthaster. (c) Diagram of experimental design for tagged Acanthaster released along each MPA border and benthic surveys conducted along each MPA border. See key below diagram for symbol identification. (TIF) [file pone.0171569.s001.tif]

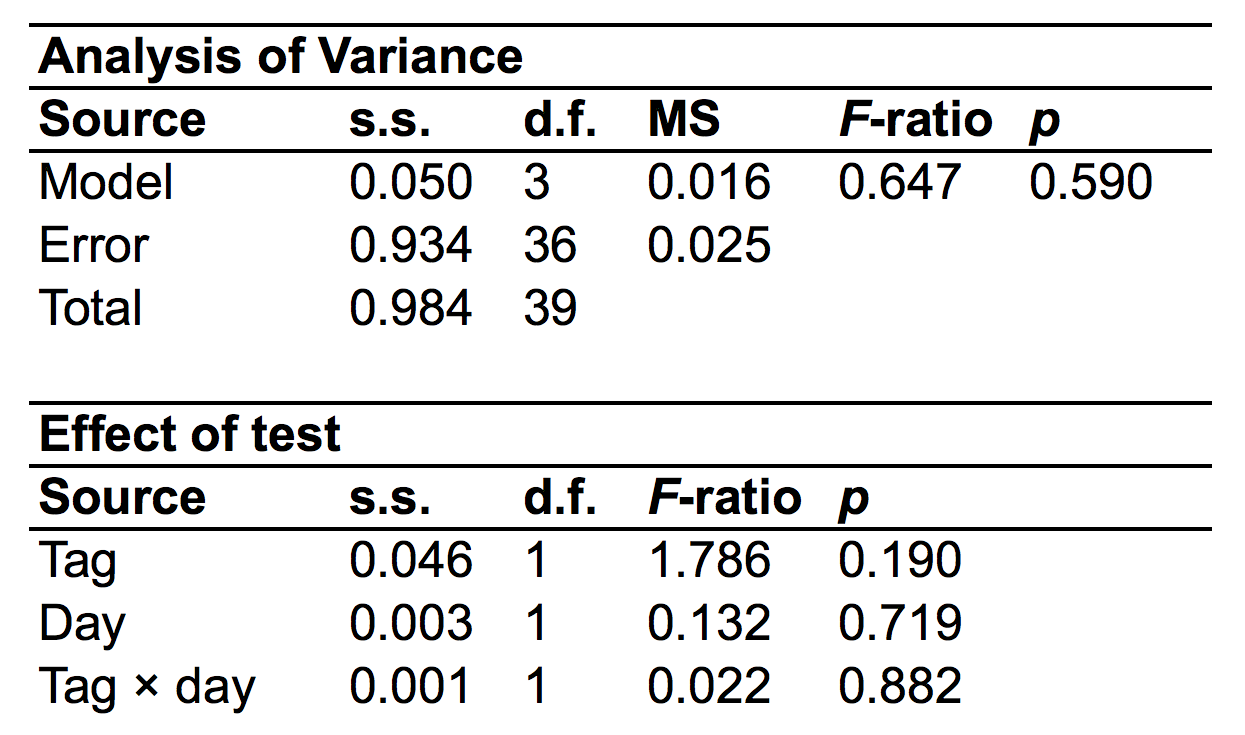

Supplement: S1 Table — Two-way ANOVA on the effect of tagging on righting time of Acanthaster after 2 and 7 days. All data were log transformed. Bartlett test for homogeneity of variances (F = 0.883, p = 0.347). (TIF) [file pone.0171569.s002.tif]

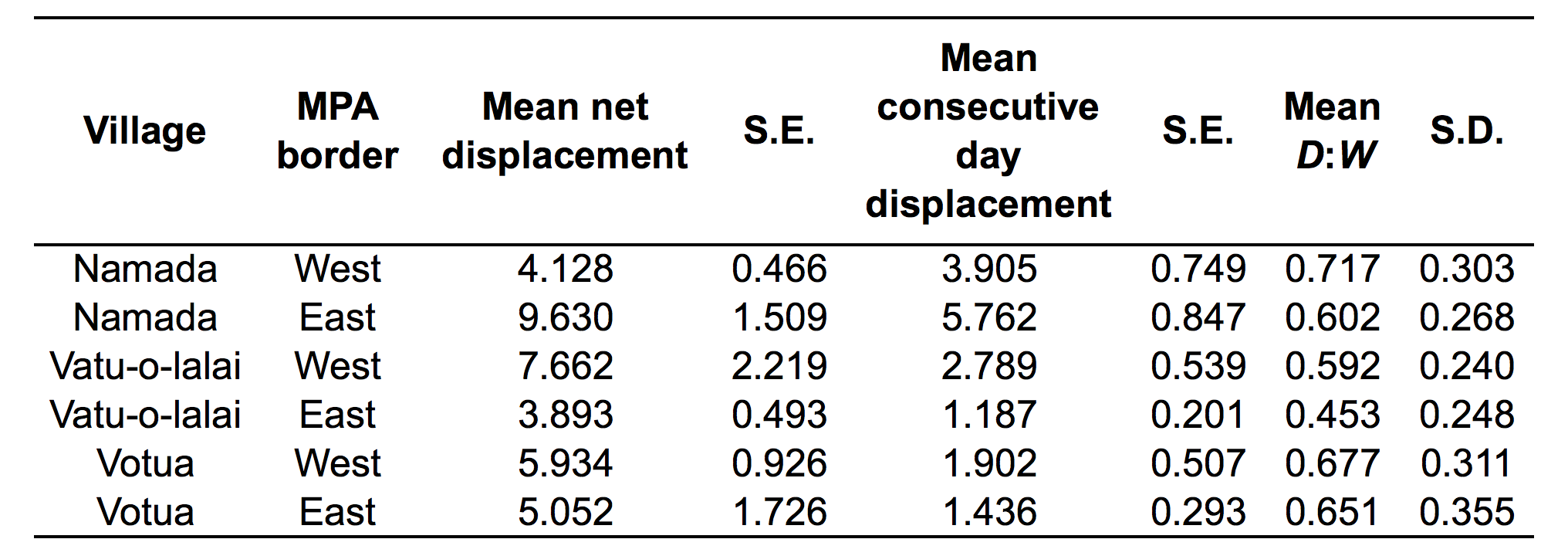

Supplement: S2 Table — Comparisons between Acanthaster of different origins tested with Fisher’s exact test. (TIF) [file pone.0171569.s003.tif]

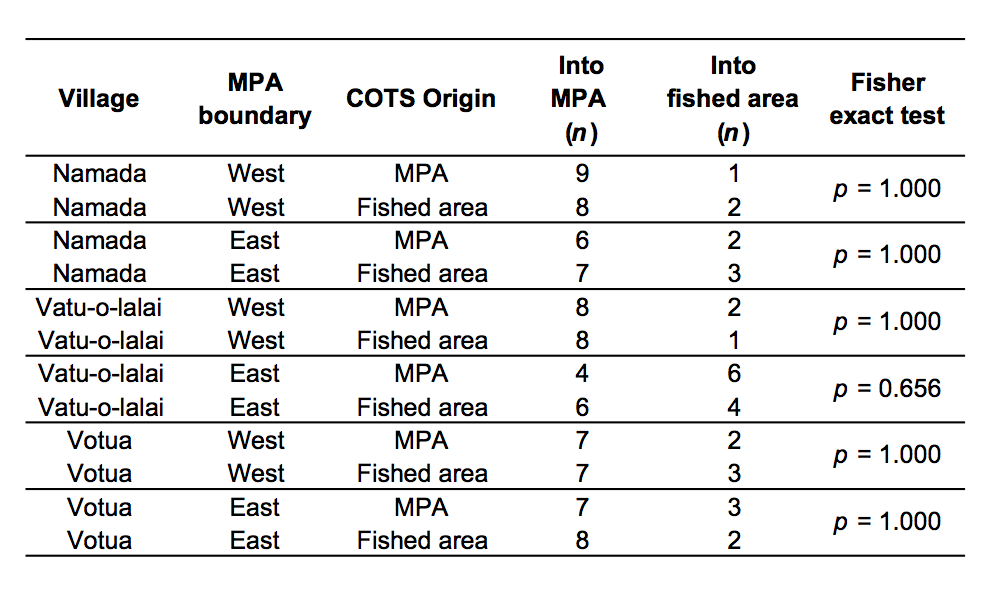

Supplement: S3 Table — Acanthaster net displacement (m day-1; mean ± SE), displacement between consecutive days (m day-1; mean ± SE), and D:W ratio (mean ± SD) at MPA border locations. (TIFF) [file pone.0171569.s004.tiff]
